# West Nile encephalitis in Israel, 1999: the New York connection.

**DOI:** 10.3201/eid0704.010410

**Published:** 2001

**Authors:** M Giladi, E Metzkor-Cotter, D A Martin, Y Siegman-Igra, A D Korczyn, R Rosso, S A Berger, G L Campbell, R S Lanciotti

**Affiliations:** Bernard Pridan Laboratory for Molecular Biology of Infectious Diseases, Tel Aviv Sourasky Medical Center, 6 Weizman Street, Tel Aviv 64239, Israel. giladi@tasmc.health.gov.il

## Abstract

We describe two cases of West Nile (WN) encephalitis in a married couple in Tel Aviv, Israel, in 1999. Reverse transcription-polymerase chain reaction performed on a brain specimen from the husband detected a WN viral strain nearly identical to avian strains recovered in Israel in 1998 (99.9% genomic sequence homology) and in New York in 1999 (99.8%). This result supports the hypothesis that the 1999 WN virus epidemic in the United States originated from the introduction of a strain that had been circulating in Israel.


### West Nile Encephalitis in Israel, 1999: The New York Connection

**Michael Giladi,* Einat Metzkor-Cotter,* Denise A. Martin,* Yardena Siegman-Igra,* Amos D. Korczyn,* Raffaele Rosso,* Stephen A. Berger,* Grant L. Campbell,† and Robert S. Lanciotti†**
*Tel Aviv Sourasky Medical Center, Tel Aviv, Israel; and †Centers for Disease Control and Prevention, Fort Collins, Colorado, USA

We describe two cases of West Nile (WN) encephalitis in a married couple in Tel Aviv, Israel, in 1999. Reverse transcription-polymerase chain reaction performed on a brain specimen from the husband detected a WN viral strain nearly identical to avian strains recovered in Israel in 1998 (99.9% genomic sequence homology) and in New York in 1999 (99.8%). This result supports the hypothesis that the 1999 WN virus epidemic in the United States originated from the introduction of a strain that had been circulating in Israel.

West Nile (WN) virus, the causative agent of WN fever and encephalitis, has a wide distribution in Africa, West Asia, and the Middle East, and outbreaks have been reported from Europe, South Africa, and Israel. Wild and domestic birds are the principal amplifying hosts of WN virus, and ornithophilic mosquitoes of the *Culex* species are the major vectors (1).

In late August 1999, the first reported outbreak of WN encephalitis in the Western Hemisphere occurred in New York City and surrounding areas. A high degree of genomic sequence similarity between virus isolates indicated that a single WN viral strain was introduced and circulated during the outbreak (2). A high (≥99.8%) genomic similarity was also found between the U.S. viral isolates and a WN virus strain isolated from the brain of a dead goose in Israel in 1998 (2).

How WN virus was introduced into the United States is not known. The high degree of similarity between the 1999 U.S. isolates and the 1998 Israeli isolate, however, raised the hypothesis that the U.S. epidemic originated from the introduction of a WN virus strain that had been circulating in Israel and surrounding countries (2). We provide more evidence to support this hypothesis.

### Case Reports

### Case 1

On August 24, 1999, a 75-year-old man was admitted to a Tel Aviv emergency room, with confusion, disorientation, and somnolence of 3 days' duration. Body temperature was 37.5°C. He was conscious but disoriented, with global aphasia. Routine laboratory test results, including cerebrospinal fluid (CSF) examination, were normal. A chest radiograph as well as electroencephalography (EEG) were normal. Computerized tomography (CT) of the brain was noncontributory. Over the next 6 days, the patient's temperature rose to 39.0°C. He became stuporous, and myoclonic jerks, as well as snout and palmo-mental pathologic reflexes, were observed. Repeat lumbar puncture revealed clear CSF with opening pressure of 160 mm $H_2O$, protein 1.36 g/L, glucose 0.6 g/L, leukocytes 120/$mm^3$ with 60% polymorphonuclear leukocytes (PMN), and 40% lymphocytes. EEG showed nonspecific, nonfocal, triphasic slow waves. Empirical treatment with acyclovir, ceftriaxone, and erythromycin was begun. During week 2 of hospitalization, the patient became less responsive, with limb spasticity, bilateral ptosis, facial nerve paralysis, and bilateral Babinski response. T2-weighted magnetic resonance imaging showed bilateral nonspecific hyperintense foci in the white matter, with lacunar changes in the striatum. Mechanical ventilation was started. Biopsy of the cerebral cortex and white matter showed reactive gliosis, isolated foci of neuronophagia, and a scanty perivascular lymphocytic infiltrate. Gradual, slow neurologic improvement was noticed starting on week 8 of hospitalization. On week 12, the patient was fully alert, with a tracheostomy but no ventilatory support. He died several months later in a rehabilitation center from bilateral pneumonia.

### Case 2

The 75-year-old wife of patient 1 was admitted to the same hospital on August 28, 1999 (4 days after her husband's admission), with fever of 39.0°C, chills, dizziness, and headache. A chest radiograph was consistent with right basilar pneumonia. Routine laboratory test results were notable only for a serum sodium level of 132 mEq/L. Empirical treatment with intravenous cefuroxime and oral roxithromycin was started. On day 4 of hospitalization, the patient became stuporous with severe respiratory acidosis; mechanical ventilation was begun. Brain CT results were normal. Lumbar puncture showed an opening pressure of 200 mm $H_2O$, protein 2.74 g/L, glucose 1.39 g/L, leukocyte count 25/$mm^3$, 80% PMN, and 20% lymphocytes. Acyclovir was added, and various antibiotic regimens were given. The patient remained febrile and stuporous and died on day 33 of hospitalization. Postmortem examination revealed mild, diffuse encephalitis involving the brain stem, and isolated

Address for correspondence: Michael Giladi, The Bernard Pridan Laboratory for Molecular Biology of Infectious Diseases, Tel Aviv Sourasky Medical Center, 6 Weizman Street, Tel Aviv 64239, Israel; fax: +972-3-697-3850; e-mail: giladi@tasmc.health.gov.il microinfarction of the basal ganglia. Bilateral pulmonary atelectasis with chronic bronchitis was also noted.

These two patients, a retired engineer and a housewife, shared an apartment in a suburb of Tel Aviv. They did not have any pets and had not left the Tel Aviv area in the preceding 5 years. They had no contact with other patients with similar clinical manifestations, nor had they entertained visitors from other countries, except their son, who had visited Germany 1 month before onset of his father's illness. An inspection by the municipal health authorities did not find mosquito infestation in the local area.

Paired CSF and serum specimens from both patients tested negative for bacteria, mycobacteria, fungi, and a large number of viruses. Results of screening tests of urine and blood for toxic substances, including botulism toxin, were also negative.

### Methods

Immunoglobulin (Ig) M-capture enzyme-linked immunosorbent assay (ELISA) and IgG ELISA were performed as described by Martin et al. (3) and Johnson et al. (4), respectively. Antigens were prepared as sucrose-acetone extracts of infected suckling mouse brains or infected C6/36 cell cultures. Positive-to-negative absorbance ratios (P/Ns) were determined by dividing the average optical density (OD) of the unknown sera by the average OD measured for the negative sera, with values >3.0 considered positive. All specimens and controls were tested in triplicate. The serum-dilution plaque reduction neutralization test (PRNT) was performed in Vero cells, as described (5). The following viruses were used: WN virus strain Eg101, dengue-2 (DEN-2) strain New Guinea C, and Japanese encephalitis (JE) virus strain Nakayama. Endpoints were determined at a 90% plaque-reduction level. A titer of 1:20 was considered a positive cutoff for PRNT results.

Fragments of brain cerebral tissue from the two patients were subjected to RNA extraction and reverse transcription-polymerase chain reaction (RT-PCR) using two different primer/probe pairs in the TaqMan assay, as described (6). For nucleic acid sequencing, the viral RNA was amplified and copied into five unique DNA fragments using the following WN/Kunjin virus primer pairs: 212/619c, 848/1442c, 1248/1830c, 9661/10,489c, and 10,571/10,815c (numbers denote positions of the primers at the Kunjin virus sequence; GenBank Accession Number D00246). DyeDeoxy Terminator cycle sequencing was performed as described (2).

### Results

Table 1 summarizes the serologic test results of both patients. In case 1, IgM antibody to WN virus was detected in serum by day 9 after onset of symptoms (P/N = 13.8). IgM was also detected in CSF on day 14 (P/N = 21.6) but not on day 3. In case 2, IgM antibody to WN virus was detected in both CSF and serum. PRNT results were positive in both cases. Patient 1 had a sixfold increase in antibody titer, 1:10 on day 9 and 1:640 on day 35 after onset of symptoms. In case 1, the positive IgM ELISA result with JE viral antigen is due to known cross-reactive antibody response to closely related flaviviruses.

The TaqMan RT-PCR assay performed on RNA extracted from the patient 1 brain biopsy specimen, obtained 33 days after onset of clinical symptoms, showed WN viral RNA when two different primer/probe sets designed from unique regions of the WN viral genome (Ct-envelope primers = 29.6, Ct-3' non-coding primers = 29.2; where Ct = threshold cycle and Ct values <37.5 are positive) were used. The quantity of viral RNA detected was 8.3 and 9.7 PFU equivalents, based on the standard curve generated in the TaqMan assay. Nucleic acid sequencing of the five RT-PCR-generated DNA fragments yielded 1,861 bp of data, approximately 17% of the total genome. Sequence comparisons demonstrated that the virus strain that infected patient 1 is most closely related to the WN-Israel 1998 strain isolated at the Pasteur Institute from a dead goose in Israel in 1998 (99.9% sequence homology; GenBank Accession Number AF205882) and to the WN-NY99 strain isolated from a dead flamingo in the Bronx Zoo, New York, in 1999 (99.8% sequence homology; GenBank Accession Number AF196835). Alignment of the sequence data revealed three positions of nucleotide differences between these three strains (positions 1,118, 1,285, and 10,851; Table 2). These nucleotide differences confirm that the WN virus strain

Table 1. Antibodies to West Nile virus and clinically related flaviviruses in two encephalitis cases, Tel Aviv, 1999

| Case 1 | Serum #1 | | | Serum #2 | | | CSF #1 | CSF #2 |
|---|---|---|---|---|---|---|---|---|
| Days[a] | | 9 | | | 35 | | 3 | 14 |
| Antigen | IgM[b] | IgG[b] | PRNT[c] | IgM | IgG | PRNT | IgM | IgM |
| WN | 13.8 | 1 | 10 | 13.7 | 3.6 | 640 | 0.9 | 21.6 |
| Den 1-4 | 2.3 | 1.2 | <10 | 2.6 | 1.7 | <10 | 1 | 1.7 |
| CHIK | 0.9 | 1.2 | | 0.9 | 1.2 | | 1.2 | 1 |
| SIN | 1 | 1.2 | | 1.1 | 1.2 | | 1.1 | 1.1 |
| POW/TBE | 1 | 1.1 | | 1.2 | 1.8 | | 0.9 | 0.8 |
| JE | 2.8 | 0.8 | <5 | 6.2 | 0.8 | 20 | 1.5 | 1.7 |
| Case 2 | | | | | | | | |
| Days[a] | | 14 | | | NA | | 7 | NA |
| Antigen | IgM | IgG | PRNT | | | | IgM | |
| WN | 13.5 | 3.1 | 80 | | | | 25.3 | |
| Den 1-4 | 1.7 | 1.3 | | | | | 1.4 | |
| CHIK | 1 | 1.4 | | | | | 0.9 | |
| SIN | 1.3 | 1.4 | | | | | 0.9 | |
| POW/TBE | 1.1 | 1.4 | | | | | 1 | |
| JE | 2.5 | 0.8 | 5 | | | | 2.1 | |

CSF = cerebrospinal fluid; CHIK, Chikungunya virus; Den 1-4 = dengue virus (types 1-4); JE = Japanese encephalitis virus; NA = not available; POW/TBE = Powassan virus/tick-borne encephalitis virus; PRNT = plaque-reduction neutralization test; SIN = Sindbis virus; WN = West Nile virus.

[a]Days = days after onset.

[b]Immunoglobulin M (IgM) and IgG antibody levels were determined by enzyme immunoassay. Results are expressed as positive-negative absorbance ratios (P/Ns), determined by dividing the average optical density of the test sera by the average optical density measured for the negative control sera, with values >3.0 considered to be positive.

[c]Results of the PRNT are expressed as reciprocal antibody titers, with values ≥20 considered to be positive.

Table 2. Nucleotide differences detected between West Nile (WN) virus genomic sequence data from patient 1, WN-Israel 1998, and WN-NY99

| WN virus nucleotide position | Sequences amplified from brain of patient 1 | WN-Israel 1998[a] | WN-NY99[b] |
|---|---|---|---|
| 1,118 | C | C | T |
| 1,285 | C | C | T |
| 10,851 | G | A | A |

[a]WN-Israel 1998 was isolated at the Pasteur Institute from a dead goose found in Israel in 1998.

[b]WN-NY99 was isolated from a dead flamingo in the Bronx Zoo, New York, 1999.

detected in the brain sample from patient 1 is not a laboratory contaminant. RT-PCR performed on an autopsy cerebral cortex brain specimen from patient 2 was negative.

### Discussion

Epidemics of WN viral disease occurred in Israel in the 1950s and in 1980 (7,8). During 1997 and 1998, WN virus was reported, for the first time, as the cause of illness and death among domestic geese in Israel. Approximately 3,000 geese with a high seroprevalence of anti-WN virus antibodies were killed to contain the epizootic (9,10). However, no human cases of WN fever were reported in Israel in 1997 to 1998 and, to the best of our knowledge, the two cases described in this report are the first and only human cases of WN fever reported in Israel in the 1990s. It seems likely that other such cases occurred in 1997 to 1999 but were unrecognized, not reported, or both.

Case 1 meets the criteria for the Centers for Disease Control and Prevention surveillance case definition of a confirmed WN encephalitis case (11). Although paired serum specimens were unavailable for case 2, the presence of WN IgM in the CSF (P/N = 25.3) and serum (P/N = 13.5) specimens obtained on day 7 and day 14, respectively, and the presence of WN virus-specific neutralizing antibodies in serum confirm this as a WN encephalitis case as well. The negative RT-PCR result on the autopsy brain specimen in case 2 is probably due to the fact that the specimen submitted for PCR was from the cerebral cortex which, on histopathologic examination, was not involved in the encephalitic process.

Several lines of evidence connect these 1999 Israeli cases with the 1999 New York WN virus outbreak. First, the Israeli and the initial American cases occurred in August 1999. Second, when genomic sequences of WN virus isolates from the New York outbreak were compared with various non-U.S. WN virus strains, the highest similarity (≥99.8%) was found with a WN virus strain from a goose that died in the 1998 Israeli epizootic (2). Similar findings were reported in another study (12). The WN virus sequences obtained by RT-PCR from a brain biopsy of the Israeli male patient shared a >99% homology with the 1999 New York and 1998 Israeli avian WN virus strains, respectively. Finally, in nature avian death caused by WN virus infection is a new phenomenon observed only in Israel and the United States (9,13).

During the summer of 2000, an epidemic of WN fever was observed in Israel, resulting in 417 serologically confirmed cases and 28 deaths (10). Several WN encephalitis cases were reported from the neighborhood of the two patients in our report. Although the genomic sequences of the isolates from 2000 are not yet available, the WN virus strain circulating in Israel since at least 1998 is likely the causative agent of the 2000 Israel epidemic as well as the 1999 New York outbreak. How this strain was transported from Israel to the United States (by infected humans, birds, mosquitoes, or other animals) remains a matter of conjecture.

Dr. Giladi is senior consultant, Infectious Disease Unit, and director, Bernard Pridan Laboratory for Molecular Biology of Infectious Diseases, Tel Aviv Sourasky Medical Center, Tel Aviv, Israel. His research interests center on the development of diagnostic tests for infectious diseases, particularly cat-scratch disease and cytomegalovirus.

### References

1. Tsai TF. Flaviviruses. In: Mandell GL, Bennett JE, Dolin R, editors. Principles and practice of infectious diseases. 5th ed. Philadelphia: Churchill Livingstone; 2000. p. 1714-36.
2. Lanciotti RS, Roehrig JT, Deubel V, Smith J, Parker M, Steele K, et al. Origin of the West Nile virus responsible for an outbreak of encephalitis in the northeastern United States. Science 1999;286:2333-7.
3. Martin DA, Muth DJ, Brown T, Johnson AJ, Karabatsos N, Roehrig JT. Standardization of immunoglobulin M capture enzyme-linked immunosorbent assays for routine diagnosis of arboviral infections. J Clin Microbiol 2000;38:1823-6.
4. Johnson AJ, Martin DA, Karabatsos N, Roehrig JT. Detection of anti-arboviral immunoglobulin G by using a monoclonal antibody-based capture enzyme-linked immunosorbent assay. J Clin Microbiol 2000;38:1827-31.
5. Beaty BJ, Calisher CH, Shope RE. Arboviruses. In: Lennette EH, Lennette DA, Lennette ET, editors. Diagnostic procedures for viral, rickettsial and chlamydial infections. 7th ed. Washington: American Public Health Association; 1995. p. 204-5.
6. Lanciotti RS, Kerst AJ, Nasci RS, Godsey MS, Mitchell CJ, Savage HM, et al. Rapid detection of West Nile virus from human clinical specimens, field-collected mosquitoes, and avian samples by a TaqMan reverse transcriptase-PCR assay. J Clin Microbiol 2000;38:4066-71.
7. Marberg K, Goldblum N, Sterk V, Jasinska-Klingberg W, Klingberg MA. Natural history of West Nile fever, clinical observations during epidemic in Israel. Am J Hyg 1956;64:259-69.
8. Katz G, Rannon L, Nili E, Danon YL. West Nile fever--occurrence in a new endemic site in the Negev. Isr J Med Sci 1989;25:39-41.
9. Israeli Ministry of Health, Jerusalem. Department of Epidemiology Quarterly Report 2000;2:6-7.
10. Israeli Center for Disease Control web site www.icdc-wnf.co.il
11. Centers for Disease Control and Prevention. Case definitions for infectious conditions under public health surveillance. MMWR Morb Mortal Wkly Rep 1997;46(RR-10):12-3.
12. Jia XY, Briese T, Jordan I, Rambaut A, Chi HC, Mackenzie JS, et al. Genetic analysis of West Nile New York 1999 encephalitis virus. Lancet 1999;354:1971-2.
13. Centers for Disease Control and Prevention. Outbreak of West Nile-like viral encephalitis: New York, 1999. MMWR Morb Mortal Wkly Rep 1999;48:845-9.
